# Glycosylation of Glycolipids in Cancer: Basis for Development of Novel Therapeutic Approaches

**DOI:** 10.3389/fonc.2013.00306

**Published:** 2013-12-19

**Authors:** Jose L. Daniotti, Aldo A. Vilcaes, Vanina Torres Demichelis, Fernando M. Ruggiero, Macarena Rodriguez-Walker

**Affiliations:** ^1^Facultad de Ciencias Químicas, Departamento de Química Biológica, Centro de Investigaciones en Química Biológica de Córdoba (CIQUIBIC, UNC-CONICET), Universidad Nacional de Córdoba, Córdoba, Argentina

**Keywords:** gangliosides, glycolipids, glycosylation, cancer, immunotherapy, antibodies, immunotoxin

## Abstract

Altered networks of gene regulation underlie many pathologies, including cancer. There are several proteins in cancer cells that are turned either on or off, which dramatically alters the metabolism and the overall activity of the cell, with the complex machinery of enzymes involved in the metabolism of glycolipids not being an exception. The aberrant glycosylation of glycolipids on the surface of the majority of cancer cells, associated with increasing evidence about the functional role of these molecules in a number of cellular physiological pathways, has received considerable attention as a convenient immunotherapeutic target for cancer treatment. This has resulted in the development of a substantial number of passive and active immunotherapies, which have shown promising results in clinical trials. More recently, antibodies to glycolipids have also emerged as an attractive tool for the targeted delivery of cytotoxic agents, thereby providing a rationale for future therapeutic interventions in cancer. This review first summarizes the cellular and molecular bases involved in the metabolic pathway and expression of glycolipids, both in normal and tumor cells, paying particular attention to sialosylated glycolipids (gangliosides). The current strategies in the battle against cancer in which glycolipids are key players are then described.

## Introduction

The aberrant and elevated expression of glycolipids has been demonstrated on the cell surface of different types of cancer cells, with these observations having opened the gate to the development of traditional immune-based treatment strategies in the battle against cancer. In general, glycolipid vaccines have failed to have a significant effect on tumor development. More recently, associated with a better comprehension about the expression, function, and membrane organization of glycolipids as well as the use of modern technologies, new immunotherapies have been developed. These therapies involve, for instance, arming monoclonal antibodies against tumor glycolipids with toxins or cytotoxic drugs; the generation of new glycolipid-specific chimeric antigen receptors in human primary T-lymphocytes; novel therapies using anti-idiotype monoclonal antibodies to sialosylated glycolipids (gangliosides); genetically engineered monoclonal antibodies to gangliosides with improved efficacy to induce antibody-mediated cellular cytotoxicity (ADCC); and complement-dependent cytotoxicity (CDC), among other original and promising immunotherapies.

Multi-institutional consortiums have been recently created to identify and quantitate all major and many minor lipids (lipidomic)^1^ and glycans (glycomic)^2^ species present mainly in mammalian cells, as well as to quantitate the changes in these species in response to perturbation. These initiatives are providing an enormous amount of information, which will certainly be of great help in the search for new targets in the treatment of cancer.

## Glycolipid Structure and Synthesis

**Figure 1:**
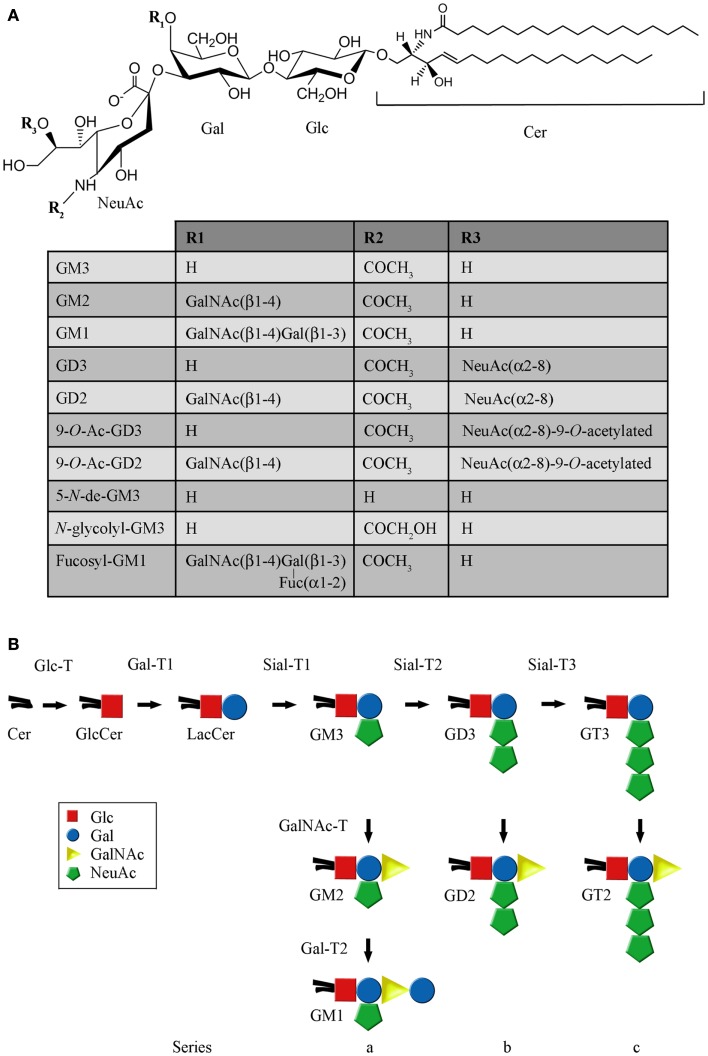
**Structure and representative reactions of ganglioside biosynthesis**. **(A)** Chemical structure of the main gangliosides mentioned in this work. **(B)** Schematic representation of the main pathway of ganglioside biosynthesis. The full names of the abbreviations are indicated in the text.

Glycolipids are lipids that have a covalently attached carbohydrate. Based on the type of lipid, they can be categorized into three main groups: glycoglycerolipids, glycosylphosphatidylinositols, and glycosphingolipids (GSLs). Mammalian GSLs begin with either glucose (GlcCer) or galactose (GalCer) attached in β-linkage to the 1-hydroxyl of ceramide (Cer) or sphingoid. GalCer can eventually be sulfated to produce acidic GSLs, referred to as sulfatides. When GlcCer is followed by addition of galactose, lactosylceramide (LacCer) is produced, which is at a branch point for formation of the root structure series (globo-, isoglobo-, lacto-, neolacto-, and ganglio-) (1). In particular, LacCer can be converted to both the neutral and acidic members of the ganglio-series by the sequential addition of different monosaccharide units, through catalytic processes mediated by type II integral membrane glycosyltransferases. Among the acidic members of the ganglio-series are found the gangliosides (Figures 1A,B), which are mono- or multi-sialosylated GSLs mainly located in the outer layer of the plasma membrane (PM) of vertebrate cells. In addition, they have been shown to be present on nuclear membranes, modulating the intranuclear calcium homeostasis (2). Gangliosides are expressed in cell-type and developmental-specific patterns, and are major components of nerve cells, where they may represent more than 10% of the total lipid content. Moreover, on the neuronal surface, they contribute more than 30% of the *N*-acetylneuraminic acid (Neu5Ac or sialic acid) (3–5).

**Figure 2:**
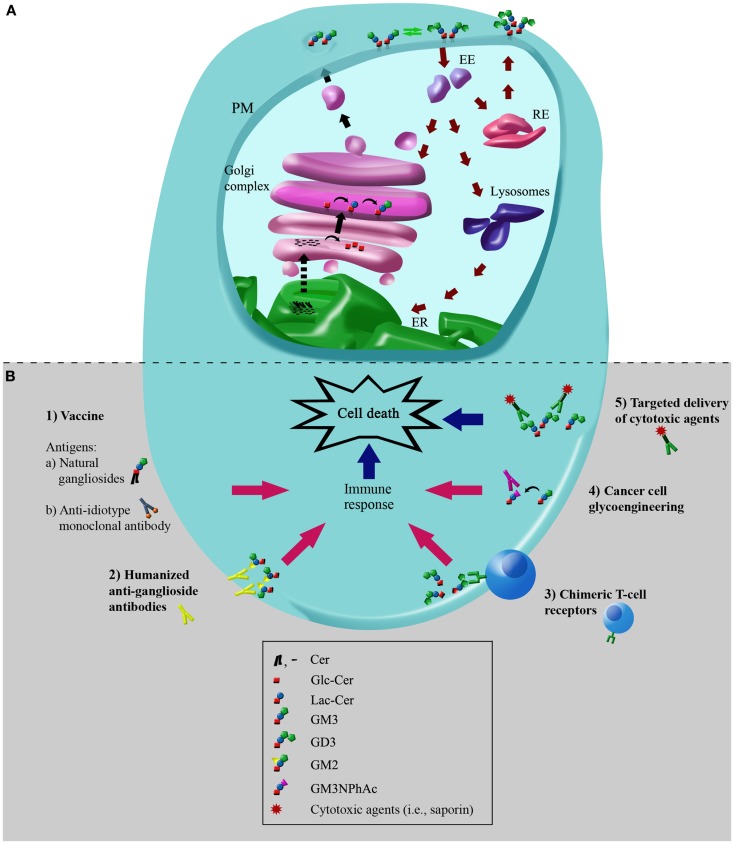
**Simplified scheme of metabolic pathways of plasma membrane-associated gangliosides: molecular targets for immunotherapies in cancer cells**. **(A)** Simplified scheme of metabolic pathways and intracellular trafficking of gangliosides. Black arrows indicate the exocytic/biosynthetic pathway. Red arrows indicate the endocytic, recycling, and catabolic pathway. Dotted arrow indicates the vesicular or protein mediated transport of ceramide between endoplasmic reticulum (ER) and the Golgi complex. Green arrows indicate remodeling of glycosphingolipids by plasma membrane (PM)-associated glycohydrolases and glycosyltransferases. The hypothetical neobiosynthesis of GM3 at the Golgi complex and later transport to PM is indicated. *De novo* synthesized gangliosides or synthesized at the PM can undergo endocytosis through clathrin-independent vesicles (caveolae), and once internalized, they can be recycled back to the PM directly from recycling endosomes (REs) or sorted from early endosomes (EEs) to the Golgi complex, where they may then be reglycosylated, or transported to the lysosomes for total or partial degradation. The representation and colors of ganglioside structures are the same as in Figure 1. **(B)** Potential cancer immunotherapies using gangliosides as molecular targets. Schematic representation depicting the main cancer immunotherapies involving gangliosides: (1) vaccination with natural gangliosides or anti-idiotype monoclonal antibodies; (2) humanized anti-ganglioside antibodies; (3) chimeric T-cell receptors; (4) cancer cell glycoengineering and monoclonal antibody-mediated selective killing of cells; (5) targeted delivery of cytotoxic agents using specific antibodies to gangliosides. See text for more details. The schematic representation and colors of gangliosides structures are the same as those indicated in Figure 1.

After synthesis of the lipophilic ceramide tail in the endoplasmic reticulum (ER), it is then transported to the Golgi complex, where it is first catalytically converted to GlcCer through UDP-Glc:ceramide glucosyltransferase (Glc-T) (Figure 2A). Then, most GlcCer can be transported back to the ER via a four-phosphate adaptor protein (FAPP2; a glycolipid-transport protein carrying a PI4P binding domain), thereby entering the secretory pathway for further conversion to LacCer in the luminal face of the *trans* Golgi and TGN (6). Other evidence indicates that ceramide can be glycosylated to GlcCer on the cytosolic leaflet of the *cis* Golgi membranes by Glc-T, and also that FAPP2 is then required for the non-vesicular transport of GlcCer to distal Golgi compartments where it then translocates for further glycosylation steps leading to more complex GSLs synthesis, which eventually includes gangliosides (7). The synthesis of LacCer occurs by the action of UDP-Gal:glucosylceramide β-1,4-galactosyltransferase (Gal-T1), which transfers a galactose residue from UDP-Gal to GlcCer (Figure 1B). Then, monosaccharide units, including sialic acid, are transferred from the cognate sugar nucleotide donor to the glycolipid acceptors produced by the transferases acting in the preceding steps in the pathway of synthesis. Sialylated derivatives from LacCer are produced by the action of cytidine monophosphate (CMP)-NeuAc:LacCer α-2,3-sialyltransferase (Sial-T1), CMP-NeuAc:GM3 α-2,8-sialyltransferase (Sial-T2), and CMP-NeuAc:GD3 α-2,8-sialyltransferase (Sial-T3), which specifically catalyze the formation of the gangliosides GM3, GD3, and GT3, respectively [ganglioside named according to Svennerholm (8)] (Figure 1B). LacCer, GM3, GD3, and GT3 are potentially converted to more complex gangliosides of the 0-, a-, b-, or c-series by sequential glycosylations catalyzed by UDP-GalNAc:LacCer/GM3/GD3/GT3 β-1,4-*N*-acetylgalactosaminyltransferase (GalNAc-T), UDP-Gal:GA2/GM2/GD2/GT2 β-1,3-galactosyltransferase (Gal-T2), CMP-NeuAc: GA1/GM1/GD1b/GT1c α-2,3-sialyltransferase (Sial-T4), and CMP-NeuAc:GM1b/GD1a/GT1b/GQ1c α-2,8-sialyltransferase (Sial-T5). These transferases are non-redundant and specific, and catalyze a sugar transfer to a glycosyl acceptor that differs only in the number of sialic acids bound to the inner galactose [none (0-series), one (a-series), two (b-series), or three (c-series)]. Exceptionally, the ganglioside GM4 (NeuAcα2,3Gal-Ceramide), a major component of the myelin, does not derive from LacCer. By the year 2004, 188 gangliosides with different carbohydrate structures had been identified in vertebrates (9).

After synthesis, gangliosides leave the Golgi complex via the lumenal surface of the transport vesicles (10). At the PM, the gangliosides can undergo endocytosis, be recycled back to the PM directly from early or recycling endosomes (REs), or sorted from endosomes at the Golgi complex, where they can then be reglycosylated or degraded at the lysosomal level (Figure 2A) (5, 11, 12).

The level of expression and diversity of GSLs, including the gangliosides, can be controlled by regulating the sugar nucleotide and acceptor availability and by enzymatic degradation, as well as through the presence and activity of the glycosyltransferases that participate in their biosynthesis. Related to this, different types of regulation of glycosyltransferases have been reported, i.e., transcriptional, translational, or post-translational (4, 13, 14). However, an additional type of regulation of GSL expression has been shown to occur at the PM level, due to PM-associated ectoglycohydrolases and glycosyltransferases (3, 15). In particular, an ectosialyltransferase (ecto-Sial-T2) recently described, it was able to sialylate GM3 exposed on its own cell or on the membrane of neighboring cells (16, 17).

## Ganglioside Function

The development of genetically engineered mice with defects in distinct biosynthetic steps of ganglioside biosynthesis has revealed the critical role played by gangliosides in a number of important biological processes, especially in the nervous system (18, 19). Gangliosides have been implicated in many physiological processes, including growth, differentiation, migration, and apoptosis through modulating both cell signaling processes and cell-to-cell and cell-to-matrix interactions (19–25). Moreover, gangliosides have been associated with a wide range of pathological processes, being receptors for viruses (i.e., simian virus 40), toxins (i.e., cholera; tetanus; and botulinum toxins), lectins, and antibodies (11, 26, 27). Some antibodies to gangliosides, in particular to GM1, GD1a, and GQ1b, have been associated with a wide range of clinically identifiable acute and chronic neuropathy syndromes, including the Guillain–Barré and Miller–Fisher syndromes (28–31), with antibodies to tumor-associated gangliosides being considered to be potential therapeutic agents, which are described below in detail (32–34).

## Ganglioside Expression in Cancer

Sialic acid-containing GSLs, gangliosides, are present in normal tissues, but they are highly expressed in many human cancer cells (Table 1). This is associated with notorious changes in the repertory of expressed species, mainly by altered glycosyltransferase and glycohydrolase activities. GD2 is a disialoganglioside involved in cell growth and differentiation, which is highly expressed on neuroblastoma, melanoma, glioma, and small-cell lung cancer (SCLC) cells (35). On the other hand, the expression level of another disialoganglioside, GD3, is very low and restricted in adult extra neural tissues. Nevertheless, GD3 is highly expressed in tumor cells, accounting for more than 80% of melanomas. It is also overexpressed in neuroectodermal tumors (neuroblastoma and glioma) and carcinomas, including lung, breast, colon, prostate, and ovary cancers (36). In addition, GD3 expression was observed in T-cell acute lymphoblastic leukemia while being absent in non-T-cell malignancies (37). For these reasons, ganglioside GD3 has received considerable attention as being a promising immunotherapeutic target for cancer therapy. In addition, GM2 is another ganglioside overexpressed in a range of cancers, including melanoma and neuroblastoma (38).

**Table 1:** **Gangliosides expressed in several types of human cancer cells**.

Type of tumor	GM3	GM2	GM1	GD3	GD2	9-*O*- Ac-GD3	9-*O*- Ac-GD2	5-N- de-GM3	Neu5Gc- GM3	Fucosyl- GM1	Reference
Melanoma	++	+		++++	+++	+	+	++^d^	++		Morton and Barth (39), Pukel et al. (40), Ravindranath et al. (41)
Neuroblastoma		++		+	++++	+					Cheung et al. (42), Hettmer et al. (43), Kohla et al. (44)
Glioma				++	++++						Mujoo et al. (35)
SCLC^a^		++	++	++	+++					+++	Brezicka et al. (45), Yoshida et al. (46, 47)
Non-SCLC	+++								+++		van Cruijsen et al. (48)
T-ALL^b^		+++		++	+						Okada et al. (49), Yamashiro et al. (50)
ATL^c^				++	++						Okada et al. (49), Yamashiro et al. (50)
Breast carcinoma	++	++		++		++			++		Marquina et al. (51)
Renal carcinoma	+	++	+								Kudo et al. (52)

*Plus signs represent ganglioside expression levels from weak (+) to strong (++++) expression*.

*^a^ Small cell lung cancer*.

*^b^ T-cell acute lymphocytic leukemia*.

*^c^ Adult T-cell leukemia*.

*^d^ 5-N-deacetylation of GM3 occurred in metastatic melanomas*.

Aberrant sialylation, both in glycoproteins and gangliosides, is closely associated with the malignant phenotypes of cancer cells, including metastatic potential and invasiveness. It was also established that the expression levels of specific neuraminidases, such as Neu1 and Neu3, are critical factors in the metastasis and survival of cancer cells, and that alteration in sialidase expression may be a defining factor for cancer progression, irrespective of the sialic acid content (53, 54).

The most common sialic acids in mammals are Neu5Ac and *N*-glycolylneuraminic acid (Neu5Gc), which are usually found as terminal constituents of different membrane glycoconjugates such as the GM3 ganglioside. Although *N*-glycolyl gangliosides are practically undetectable in normal human tissues as a result of an *Alu*-mediated inactivation of the gene coding for the enzyme CMP-NeuAc hydroxylase, these gangliosides are highly expressed in several human cancer cells (i.e., non-SCLC; Table 1) presumably due to incorporation of dietary Neu5Gc (55, 56). Furthermore, it has been proposed that the preferential expression of Neu5Gc in cancers is closely associated with tumor hypoxia, which induces expression of a sialic acid transporter and enhances the incorporation of non-human sialic acid from the external milieu (57).

GD3 ganglioside can undergo 9-*O*-acetylation at C9 of the outer sialic acid (9-*O*-Ac-GD3) with enhanced expression of the 9-*O*-acetylated form of GD3 ganglioside having been demonstrated in malignant melanomas and in basal cell carcinomas (58–60). Additional evidence also indicates that whereas GD3 enhances apoptosis, 9-*O*-acetyl-GD3 has the opposite effect (61).

Recently, it has been reported that 5-N-deacetylation of GM3 (5-N-de-GM3), specifically expressed in metastatic melanomas, but not in normal tissues or in the majority of primary melanomas or benign nevi, correlates with an enhanced metastatic phenotype. Furthermore, it has also been demonstrated that 5-N-de-GM3 stimulates cell migration and invasion by increasing the expression and activation of urokinase-like plasminogen activator and matrix metalloproteinase-2. Thus, 5-N-de-GM3 can be considered as a specific marker for metastatic melanoma and as a promising immunotherapeutic target for cancer therapy (62).

Fucosyl-GM1 is a ganglioside with a unique structure in which the terminal galactose is α-1,2-fucosylated at the non-reducing end. It is expressed in very few normal tissues but occurs in a variety of cancers such as in SCLC. Consequently, fucosyl-GM1 has also been considered to be a candidate as a tumor marker and target antigen in antibody immunotherapy (63, 64).

## Tumor-Associated Gangliosides: Molecular Targets for Passive and Active Immunotherapies

The use of antibodies to specifically target different cell populations has become a desirable method for treatment of a variety of diseases, including cancer. In fact, cell surface receptors are the main targets for immunotherapy due to the fact that they often play important roles in tumor biology, where they are overexpressed or display abnormal signaling. Basically, immunotherapeutic strategies include the use of two types of immunization, passive and active, with passive immunity being the transfer of humoral immunity in the form of ready-made antibodies from one individual to another (natural), or achieved by the artificial transfer of antibodies that can be administered in several forms (human or animal plasma or serum, such as pooled human immunoglobulin or monoclonal antibodies). Passive immunotherapeutic can also include the use of unlabeled antibodies, radiolabeled antibodies, or antibody-drug conjugates, which is describe below. On the other hand, active immunization is the induction of humoral or cellular immunity after exposure to an antigen, which can occur both naturally and artificially.

Glycolipids in general and gangliosides in particular, have received considerable attention as a convenient immunotherapeutic target for cancer treatment. This has resulted in the development of a substantial number of potential passive and active immunotherapies (Table 2; Figure 2B), of which some are briefly described below.

**Table 2:** **Immunotherapeutic strategies involving tumor associated gangliosides**.

Ganglioside	Type of tumor	Type of treatment	Type of acquired immunity	Reference
GM3	Melanoma	mAb anti-GM3NPhAc 2H3	Passive	Pan et al. (65)
		Glycoengineered GM3NPhAc-KLH and ManNPhAc	Active	Qiu et al. (66), Wang et al. (67)
		*N*-glycolyl GM3 ganglioside vaccine	Active	Carr et al. (68), Osorio et al. (69)
		Anti-idiotype mAb (racotumomab)	Active	Vazquez et al. (57, 70)
	Bladder cancer	Addition of GM3	Passive	Wang et al. (71)
GM2	Lymphoma	mAb DMF10.167.4 (*in vitro*)	–	Fernandes et al. (72)
	Melanoma and SCLC	mAb DMF10.167.4	Passive	Retter et al. (73)
	Multiple myeloma and SCLC	Hu-mAb BIW-8962 and KM8927	Passive	Yamada et al. (74), Richardson et al. (75)
	Melanoma	GM2-KLH/QS-21 vaccine	Active	Slovin et al. (76), Eggermont et al. (77)
GD3	Melanoma	mAb R24	Passive	Nasi et al. (78)
			Active	Ravindranath and Morton (79)
		Anti-GD3 chimeric sFv-CD28/T-cell	Passive	Lo et al. (36)
		mAb anti-idiotype (BEC2)	Active	Grant et al. (80), McCaffery et al. (81)
		R24 anti-anti-idiotype mAb	Passive	Ramos et al. (33)
		mAb R24-saporin (*in vitro*)	–	Torres Demichelis et al. (32)
GD2	Melanoma	Chimeric 14.18 Ab-IL-2 (*in vitro*)	–	Gillies et al. (82)
		Hu-mAb L72	Passive	Irie and Morton (83)
		Immunotoxin 14.G2a mAb-ricin A (*in vitro*)	–	Wargalla and Reisfeld (84)
		mAb anti-idiotype (1A7)	Active	Sen et al. (85, 86), Foon et al. (87, 88)
		Immunocytokine chimeric 14.18 mAb-IL-2	Passive	Becker et al. (89)
	Neuroblastoma	M-mAb 3F8/Hu-mAb 3F8	Passive	Irie and Morton (83), Cheung et al. (90, 91), Kushner et al. (92)
		M-mAb 14.G2a/M-mAb 14.G2a + IL-2	Passive	Mayer et al. (93), Frost et al. (94), Handgretinger et al. (95)
		Immunotoxins 14.G2a mAb-ricin A/BW704dgA	Passive	Gottstein et al. (96), Manzke et al. (97)
		14.G2a chimeric T-cell receptors	Passive	Rossig et al. (98), Pulè et al. (99)
		Anti-idiotype mAb (ganglidiomab)	Active	Lode et al. (100)
		Immunocytokine chimeric 14.18 mAb-GM-CSF (*in vitro*)	–	Batova et al. (101)
		Immunocytokine chimeric 14.18 mAb-IL-2	Passive	Sabzevari et al. (102)
Fucosyl-GM1	SCLC	mAb F12 and F15	Passive	Brezicka et al. (103)
		Fucosyl-GM1-KLH vaccine	Active	Dickler et al. (104)
9-*O*-Ac-GD2	SCLC, lymphoma, neuroblastoma, ovarian carcinoma	mAb 8B6	Passive	Alvarez-Rueda et al. (105)

*mAb, monoclonal antibody; M, mouse; Hu, human*.

## Immunotherapies Using Ganglioside GM3 as the Target

GM3 can be defined as a tumor-associated carbohydrate antigen, since it is significantly overexpressed by a number of tumors, such as melanoma (39), and expressed at lower concentrations in many normal cells (106). It has been demonstrated that unnatural *N*-acyl derivatives of GM3, especially *N*-phenylacetyl GM3 (GM3NPhAc), were much more immunogenic than native GM3, and that GM3NPhAc could provoke a robust T-cell dependent immune response in mice (65), which is critical for the anti-tumor activities of a cancer immunotherapy. More recently, some studies have demonstrated an efficient metabolic glycoengineering of GM3 on melanoma cells with monoclonal antibody-mediated selective killing of glycoengineered cancer cells. Basically, cells were metabolically labeled both *in vivo* and *in vitro* with *N*-phenylacetyl-d-mannosamine (ManNPhAc) and then a monoclonal antibody (2H3), which recognizes both GM3NPhAc and ManNPhAc, was employed to selectively target and kill metabolically glycoengineered cancer cells (66, 67).

The immunogenic and toxicity profile of the heterophilic Neu5Gc-GM3 ganglioside vaccine in patients with advanced breast cancer was previously described. This vaccine, which is formulated by the combination of gangliosides with the outer membrane protein of *Neisseria meningitides* to form very small size proteoliposomes (VSSPs), resulted in acceptable safety outcomes. In addition, this technology permits an active immunotherapy that involves activation of the potent innate natural immune system (68). More recently, a Phase Ib/IIa clinical trial was carried out in patients with advanced cutaneous and ocular malignant melanomas in order to evaluate the immunogenicity and toxicity of an intramuscularly administered cancer vaccine composed of Neu5Gc-GM3/VSSP (69). The results obtained indicated the safety and immunogenicity of the vaccine and reinforced the position of gangliosides as targets for immunotherapy.

An attractive approach to generate an effective immune response against tumor-associated antigens involves the use of an anti-idiotype monoclonal antibody and appropriately selected anti-idiotypic antibodies that can act as tumor-associated antigen substitutes. As already mentioned above, although *N*-glycolyl gangliosides are practically undetectable in normal human tissues, these gangliosides are highly expressed in several human cancer cells. For example, racotumomab is an anti-idiotype monoclonal antibody specific to an antibody, which reacts to Neu5Gc-containing gangliosides, sulfatides, and other antigens expressed in tumors. This antibody was able to induce a strong anti-metastatic effect in tumor-bearing mice (70), and more recently, a Phase II/III multicenter double-blind clinical trial was conducted to evaluate the effects of racotumomab vaccine in the overall survival in patients with advanced non-SCLC. The results of this study showed a significant clinical benefit in the patients who were treated with the anti-idiotype vaccine (57).

## Immunotherapies Using Ganglioside GM2 as the Target

Ganglioside GM2 is involved in cell adhesion and signal transduction, and it has also been reported to play a role in tumor metastasis. As normal cells express little GM2, it is an ideal target for anti-metastatic therapy. Related to this, DMF10.167.4 is a hamster monoclonal antibody raised against a murine T-cell lymphoma cell line and has been shown to induce apoptosis *in vitro* (72). This antibody was found to react with a GM2 epitope that is expressed on a large number of tumor cell lines, including human melanoma and SCLC, but not on normal primary lines or most normal tissues (73). It was shown to have immunotherapeutic potential, since it was able both to prevent tumors being established *in vivo* and to block the progression of established tumors.

The anti-metastatic effects of the two humanized anti-ganglioside GM2 antibodies, BIW-8962 and KM8927, have been recently investigated and compared with the chimeric anti-GM2 antibody KM966, in a mouse model of multiple organ metastases induced by GM2-expressing SCLC cells (74). These humanized antibodies inhibited the production of multiple organ metastases, increased the number of apoptotic cells, and prolonged the survival of the mice, which suggests that humanized anti-GM2 antibodies may be therapeutically useful for controlling multiple organ metastases of GM2-expressing SCLC.

GM2 ganglioside has also been used for vaccination in combination with a T-cell carrier such as keyhole limpet hemocyanin (KLH), or with other adjuvants such as purified mycobacterial cell-wall skeleton or QS-21, a saponin-based adjuvant. The GM2-KLH vaccine not only induced a IgM response, but also induced durable IgG antibodies in most patients in early clinical trials (76). Nevertheless, a recent study shows that GM2-KLH/QS-21 vaccination does not improve the outcome for patients with stage II melanoma (77).

## Immunotherapies Using Ganglioside GD3 as the Target

The ganglioside GD3 is a glycolipid highly expressed during the early developmental stages of the central nervous system, when the neural cells proliferate actively. At later developmental stages, the GD3 content declines and other gangliosides become more abundant (107, 108) with the expression level of GD3 being very low and restricted in adult extra neural tissues. However, GD3 is highly expressed in tumor cells in more than 80% of melanomas. It is also overexpressed in neuroectodermal tumors (neuroblastoma and glioma) and carcinomas (36). For these reasons, ganglioside GD3 has received considerable attention as a promising immunotherapeutic target for cancer therapy. As such, it has been used for passive (78) and active (79) immunotherapy of melanoma cancer. However, the results obtained with this antibody therapy are modest (76), and the generation of new GD3-specific chimeric antigen receptors with improved efficacy in human primary T-lymphocytes is currently being evaluated (36).

Many studies have also involved the use of anti-idiotype vaccine in cancer patients, which mimics GD3 ganglioside (109). In particular, the monoclonal antibody Bec2 has been studied in melanoma and SCLC patients, where it induced specific anti-GD3 antibodies, but only in a low percentage of patients (80, 81). Later, a Phase III clinical trial with 515 SCLC patients revealed a major response to chemotherapy and chest radiation with this antibody. Although this trial failed to show any survival advantage for vaccinated patients, a trend toward prolonged survival was observed in those patients who developed the humoral response (110).

The mouse monoclonal R24 antibody (IgG3), directed against ganglioside GD3, is a validated tumor targeting agent that has shown strong cell surface reactivity for a range of human melanoma cell lines and other epithelial tumor cancer cells (40). It was demonstrated in our laboratory that, for different cell lines, the R24 antibody to GD3 was rapidly endocytosed after binding to the disialoganglioside at the cell surface before being sorted to early endosomes and later accumulated in REs (111). Therefore, its rapid internalization in cells precludes its use as a “naked therapeutic antibody,” because when internalized it cannot link to pathways of complement- or cellular-dependent anticancer activities. However, we took advantage of the internalization feature of R24 antibody for selective delivery of the toxin saporin (a ribosome-inactivating protein) to GD3-expressing cells (32). This was carried out using a goat anti-mouse IgG antibody linked to the ribosome-inactivating toxin, and additionally, biotinylated R24 antibody was used for targeted delivery of streptavidin-saporin. This immunotoxin was found to be specifically cytotoxic for GD3-expressing cells [human (SK-Mel-28) and mouse (B16) melanoma cells and Chinese hamster ovary-K1 cells] grown on 2D monolayers and for cells grown in attachment-free conditions. Thus, ganglioside GD3 emerge as a novel and attractive class of cell surface molecule for the targeted delivery of cytotoxic agents, and therefore, provides a rationale for future therapeutic intervention in cancer.

## Immunotherapies Using Ganglioside GD2 as the Target

GD2 is a disialoganglioside normally expressed on skin melanocytes, neurons, and peripheral nerves. It is overexpressed in cancers such as neuroblastoma, melanoma, glioma, some types of non-SCLC (112) and also in sarcomas (64), and thus is a promising target to treat these types of cancers. In fact, targeting GD2 has the important advantage that is not shed by cells into the microenvironment (113), in contrast with other studied gangliosides (114). Many antibodies that target this ganglioside have been studied, with Hu-mAb L72 being shown to react specifically with ganglioside GD2 and have a strong cytotoxic effect on human melanoma cells in the presence of complement (83). The 3F8 antibody has been assessed in clinical trials in patients with neuroblastoma (90, 92). Humanizing m3F8 produced anti-GD2 antibodies with an increased ADCC potential *in vitro* and anti-tumor activity *in vivo*, also reduced pain and human anti-mouse antibody side effects (91). However, in a recent report, posterior reversible encephalopathy syndrome (PRES) in neuroblastoma patients receiving anti-GD2 3F8 was documented (115). The use of 14.G2a mouse monoclonal antibody showed granulocyte mediated ADCC in neuroblastoma cells *in vitro* (93), and when administrated to patients concomitantly with IL-2 (94).

When the effect of an immunotoxin covalently linking ricin A chain toxin to 14.G2a mouse monoclonal antibody was evaluated, cytotoxic activity of this immunocomplex against human tumor cells was reported *in vitro* (84) and in a disseminated human neuroblastoma in a severe combined immunodeficiency (SCID) mice model (96). In addition, it was described the antitumoral capacity of two immunotherapeutic approaches using GD2 binding BW704 antibody conjugated to deglycosylated ricin A and to an anti-CD3 × anti-GD2 bispecific antibody that was capable of redirecting cytotoxic T-cells toward neuroblastoma cells. The results obtained *in vitro* and *in vivo* suggest a potential role of these immunotherapeutic agents in the treatment of minimal residual disease in the advanced stages of neuroblastoma (97).

Anti-idiotype antibodies have also been investigated with respect to their ability to overcome difficulties and activate an appropriate immune response when GD2 ganglioside is used as immunogen. In one study, ganglidiomab was generated following immunization of Balb/c mice with 14G2a and splenocytes were then harvested to generate hybridoma cells. It was demonstrated induction of a GD2-specific humoral immune response after vaccination of mice with ganglidiomab effective in mediating GD2-specific killing of neuroblastoma cells (100). In other investigations, the murine monoclonal anti-ganglioside GD2 antibody 14.G2a was used to generate the monoclonal anti-idiotype 1A7 (85) which has been used as an anti-idiotype vaccine in patients with advanced melanoma (87, 88). Immunization with this anti-idiotype vaccine elicited a strong anti-GD2 antibody response that specifically reacted with tumor cells expressing GD2 with the results suggesting that this anti-idiotype vaccine may have a role in preventing disease progression and in increasing the survival time for patients with advanced malignant melanoma (87).

Antibody-cytokine fusion proteins, designed to achieve optimal biological effectiveness by combining the unique targeting ability of antibodies with the multifunctional activities of cytokines (82, 116), have been also used as cancer vaccine. In addition, immunocytokines have been generated by fusion of a human/mouse chimeric anti-ganglioside GD2 antibody (ch14.18) with cytokines GM-CSF and IL-2, and proved to be effective in killing GD2 positive cells *in vitro* by CDC and ADCC mediated by granulocytes (101). The ch14.18-IL-2 fusion protein was also shown to have provided anti-tumor effects in SCID mice bearing human tumor xenografts of neuroblastoma (102) and melanoma (89).

9-*O*-Ac-GD2 ganglioside is a modified GD2 ganglioside which is expressed in neuroblastoma, SCLC, melanoma, and renal carcinoma, but not in peripheral nerve fibers, ovarian carcinoma, or pancreatic carcinoma (105). A monoclonal antibody to *O*-acetyl-GD2, 8B6, was developed and showed (both *in vitro* and *in vivo*) anti-tumor properties that were comparable to those elicited by anti-GD2 14.G2a. More recently, it was further demonstrated that the 8B6 antibody was very effective in suppression of tumor growth in mice by reducing the cell proliferation index and inducing apoptosis. Moreover, it was also observed that the lytic function of NK cells and complement were not a requirement for the *in vivo* activity of the 8B6 antibody (117).

## Immunotherapies Using Ganglioside Fucosyl-GM1 as the Target

Fucosyl-GM1 is a characteristic glycolipid expressed in SCLC cells, which is detected with high frequency in SCLC in comparison with other cancers of the lung or in normal lungs or bronchus (with it only being sporadically expressed, if at all, in normal tissues) (45, 64). However, the immune system does not seems to be able to respond against this fucosylated glycolipid, as there is no production of auto-antibodies (118). The fact that it is an exclusively antigen expressed on SCLC cells turns it into an appropriate candidate for therapy. Related to this, two monoclonal antibodies against fucosyl-GM1 (F12 and 15) induced CDC and ADCC in fucosyl-GM1 expressing cell lines *in vitro*, and also conferred protection against tumor engraftment in a mouse model (103). In clinical trials of immunization with fucosyl-GM1-KLH conjugate, SCLC patients demonstrated serological IgM and IgG responses against fucosyl-GM1 and *in vitro* CDC of fucosyl-GM1 positive cell lines (104). When the conjugate was produced using synthetic fucosyl-GM1 (119), the IgM response and *in vitro* CDC were comparable to those obtained with the natural fucosyl-GM1 conjugate but without the IgG response. Finally, in a recent study, it was also suggested as a potential marker of hepatocellular carcinoma (120).

## Concluding Remarks

Over recent decades, a substantial number of cancer immunotherapies have been developed, that have used the sialosylated glycolipids as the main target (see Table 2; Figure 2B). Although many of these therapies have failed to result in a significant effect on tumor development, others have led to promising results. We have learnt about how we can address an immune response to a specific cell surface ganglioside, but certainly, there is still much to do in terms of improving these immunotherapies. With the advent of modern technologies, new and combinatorial immunotherapies are currently being developed in an attempt to overcome some of the limitations of using glycolipids as vaccine antigen, such as their poor immunogenicity, low-affinity immunoglobulin responses, and immunotolerance. In this sense, mimetic peptides represent a very promising tool to overcome T-cell independence of some carbohydrate antigens. The development of DNA vaccine encoding designed peptide mimotope (minigene) of GD2 ganglioside has been demonstrated to be effective in inducing protective GD2 cross-reactive IgG antibody responses (121, 122). As also proposed, engineering the mimotope into a hybrid plasmid, which can also include cytotoxic T-lymphocyte epitopes from a tumor target itself, would be expected to build an effector response that could improve the tumor-protective immunity evoked by the minigene vaccine (121). Finally, studies are also being focused on the development of new strategies for therapeutic intervention in cancer, which propose the use gangliosides for targeted delivery of cytotoxic agents via specific antibodies (32), or eventually, via small-molecule cyclic peptide ligands (123).

## Authors Contribution

Jose L. Daniotti, Aldo A. Vilcaes, Vanina Torres Demichelis, Fernando M. Ruggiero, and Macarena Rodriguez-Walker contributed to the conception and design of the work. All authors wrote, edited, and reviewed the final manuscript version.

## Conflict of Interest Statement

The authors declare that the research was conducted in the absence of any commercial or financial relationships that could be construed as a potential conflict of interest.

## Footnotes

^1^http://www.lipidmaps.org

^2^http://www.functionalglycomics.org
